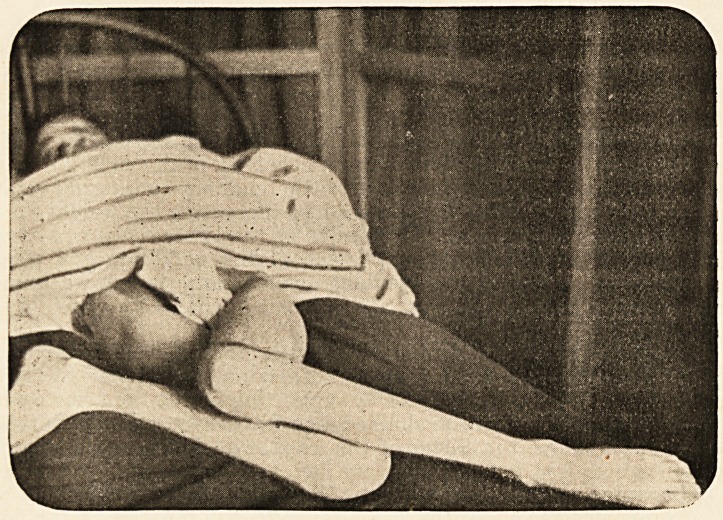# A Case of Hysterical Disease of Both Hips and Both Knees, with Extreme Distortion of the Lower Limbs

**Published:** 1897-03

**Authors:** Charles A. Morton

**Affiliations:** Surgeon to the Bristol General Hospital


### A CASE OF HYSTERICAL DISEASE OF BOTH HIPS AND BOTH KNEES, WITH EXTREME DISTORTION OF THE LOWER LIMBS.

BY

CHARLES A. MORTON, F.R.C.S. Eng.,
*Surgeon to the Bristol General Hospital.*

THE following case is a remarkable instance of the mimicry of joint-disease in hysteria. For three years the patient was under the treatment of various surgeons for organic disease of the hip, and yet when the true nature of the affection was recognised she was cured in less than three months. The affection for more than two years was confined to one hip, and was considered to be certainly tubercular in nature, and the treatment adopted was the ordinary one for such a condition—absolute rest, with splint and extension. Moreover, repeated examination of the joint under an anæsthetic failed to reveal its true nature.

V. J., aged 19, came under my care at the Bristol General Hospital on November 1st, 1896. When 9 years of age she had been in a large provincial hospital with "disease in the left hip-joint." She had extension applied for three months, but no splints, and then recovered. When 12 years of age she was re-admitted for the same trouble, and then had a long splint and extension applied for six months, and at the end of that period was sent home in a Thomas splint. The third attack came on when she was 16, with pain in the same joint, which prevented her walking. At first the limb was not drawn up or fixed in any abnormal position, but it soon became so, and she was an in-patient at the same hospital (where she had been as a child) during the years 1894 and 1895, and was supposed to be suffering from tubercular disease of the left hip-joint. Twice the limb was straightened under chloroform, and a splint and extension kept constantly applied. She was sent home in a Thomas splint in January, 1896. Two months after she returned home pain set in in the right hip and in both knees, and the right lower limb became drawn up, and both more deformed. A suspicion of phthisis at the right apex tended to strengthen the opinion of her medical attendant that the disease in the joints was tubercular. In April, 1896, the left lower limb was straightened under chloroform and put up in a long splint with extension, but the splint was removed later and the limb became again distorted.

When she came under my care her limbs presented the appearance shown so well in this photograph, taken by my dresser, Mr. Aubrey.

So great was the distortion that it seemed marvellous that the limbs could assume such a position, and I had difficulty in persuading some of those who saw her that there was no actual dislocation of the right hip. The position which the limb assumed was not possible with a normal hip, and the extreme rotation inwards, which enabled the inner side of the leg to lie flat on the bed in the flexed position of the knee, could only have been gradually produced by ever-increasing relaxation of the ligaments of the hip. The position of the limbs may be thus described: Marked flexion of both hips, obscured by lordosis, and thus not represented in the photograph. Extreme adduction of both thighs, with internal rotation. The internal rotation of the right was so great that the trochanter lay in a vertical line with the anterior-superior spine. The left knee was flexed to a right angle, and the right was much more flexed. Both hips and knees were absolutely fixed, and any attempt to move them gave rise to expressions of great pain. The muscles of the limbs, especially the left, were greatly atrophied, and consequently the articular ends of the bones were apparently enlarged, but were not really so; neither was there any sign of pulpy thickening about the knees or of deep swelling about the hips, nor of increased heat over the joints. Moreover, grasping the tuberosity of the ischium, or other fixed point of bone, gave rise to as much pain as did any attempt to move one of the affected joints. It also seemed to me quite unlikely that, if tubercular, the disease could have existed in the left hip-joint in an active form for three years without leading to abscess.

She had what I can only describe as a neurotic expression, but there were no other manifestations of hysteria in the form of local anæsthesia or globus hystericus, nor was there any family history of neurotic ailments.

I decided to treat her for hysterical disease. On November 5th chloroform was administered. Directly she went under, the muscles of the lower limbs relaxed, and they fell into a much more normal position, and manipulation easily rectified the remaining deformity. No grating was discovered in any joint. A few adhesions gave way in the knee-joints on fully extending them, and the ligaments of these joints were found so relaxed that lateral movement of the articular surfaces was found possible to a considerable extent. I applied short back splints behind both knees to prevent flexion, and put 5 lbs. extension on each lower limb to prevent flexion or adduction of the thighs.

She did not suffer much pain in the joints after she came out of the anæsthesia. A little painless effusion occurred in the right knee, and lasted for a fortnight. There was a little tendency to internal rotation of the right hip, but this was rectified by increasing the weight on that limb to 7 lbs. Massage of the muscles of the lower limbs was at once commenced. She took a hopeful view of her condition and seemed to believe she would soon be walking about again, though we found it impossible to persuade the mother that this might be so. She was not isolated.

A month after the administration of the chloroform I removed the splints and moved all the joints. I then persuaded her to try and move them, and she did so, and by supporting her by the arms we held her up on the floor and she shuffled her feet along. The splints were not re-applied, but the Sister of the ward, who had a most beneficial influence over her, got her up every day, and she soon began to walk. The massage was continued, and by the end of the year she could walk slowly but steadily about the Hospital, and the muscles of the lower limbs had considerably increased in bulk. The flail-like condition of the right knee had passed off, but there was still some lateral motion possible in the left. There was no pain in any joint and no tendency to distortion. She was discharged on January 23rd.

One difficulty which presented itself in the treatment of this case was to prevent recontraction of the limbs after the straightening under anæsthesia, on the one hand, and the possible fixation of the limbs in the straight position on the other. I felt that if we did not fix the limbs in the straight position, but tried to get her to stand and walk directly she recovered from the effects of the anæsthetic, recontraction was exceedingly likely to occur, and then all further attempts would be impossible; whereas if her limbs did become rigid in the straight position, we could still put her on her feet and encourage her to make an effort to walk. Happily no rigidity set in while the splints and extension were employed, but the muscles began to develop with the massage, and the result was most satisfactory. Had we been less fortunate, I should certainly have insisted on isolation. The relaxation of the ligaments and the slight adhesions in the knee-joints were certainly the result of the long-standing extreme deformity produced by muscular contraction and the fixation of the joints. There seems to me little doubt that the two earlier attacks of "hip disease" were also hysterical. They were in the same hip, first and for so long affected in the third attack.

## Figures and Tables

**Figure f1:**